# Lactoferrin in the Prevention of Recurrent Respiratory Infections in Preschool Children: A Prospective Randomized Study

**DOI:** 10.3390/children11020249

**Published:** 2024-02-15

**Authors:** Angela Pasinato, Mario Fama, Giovanni Tripepi, Colin Gerard Egan, Eugenio Baraldi

**Affiliations:** 1 Società Italiana Cure Pediatriche Primarie (SICuPP), Veneto Region, 20126 Milano, Italy; angela.pasinato@aullss8.veneto.it (A.P.); dott@mariofama.it (M.F.); 2 National Research Council (CNR), Ospedali Riuniti, 89124 Reggio Calabria, Italy; giovanniluigi.tripepi@cnr.it; 3 CEMW SRLS, 56021 Pisa, Italy; cegan@ce-medicalwriting.com; 4 Dipartimento di Salute della Donna e del Bambino, Azienda Ospedale-Università di Padova, 35128 Padova, Italy

**Keywords:** recurrent respiratory infections, preschool children, lactoferrin, corticosteroids, real-life

## Abstract

Few studies have evaluated the effect of bovine lactoferrin (bLf) on reducing respiratory infections in preschool children. This randomized controlled trial evaluated the effect of bLf in preschool children with recurrent respiratory infections. Participants were randomly assigned bLf (n = 25) or control (*n* = 25). Outcomes included respiratory infection episodes (RIEs), symptom duration, school absence and medication. Fifty children aged 4.2 ± 0.1 years were included. During the active 4-month phase, median number of RIEs was reduced by 50% in the bLf group [1-episode, interquartile range (IQR): 0–2] vs. control (2, IQR: 1–3; *p* = 0.02). The proportion of participants with >3 RIEs was significantly lower in bLf (*n* = 1, 4%) vs. control (*n* = 7, 28%) with 80% lower odds of upper RIEs in the bLf arm (odds ratio: 0.20, 95% CI:0.06–0.74, *p* = 0.015). The duration of symptoms (3 vs. 6, *p* = 0.009) and days absent from school (3 vs. 6, *p* = 0.15) were lower in the active arm. Over the 2-month follow-up, no significant differences were observed between groups for infection episodes, symptom duration or school absence. However, bLf-treated children received significantly less corticosteroids over the entire 6-month study period (32% vs. 60%; *p* = 0.047). bLf supplementation significantly reduced the frequency and duration of RIEs in children with decreased corticosteroid use.

## 1. Introduction

Respiratory tract infections (RTIs) can affect either the upper or lower respiratory tract, with associated symptoms such as fever, general malaise, cough, sore throat, ear pain, nasal congestion, rhinorrhea, and wheezing. Upper respiratory tract infections include rhinitis, pharyngitis, tonsillitis, laryngitis, and middle ear infections (otitis media). Lower respiratory tract infections, including tracheitis, bronchiolitis, bronchitis, and pneumonia, can have severe complications requiring hospitalization [1]. The majority of RTIs are of viral origin, with bacterial causes being less frequent. In 80% of cases, the culprits behind these infections are rhinoviruses, parainfluenza viruses (types 1, 2, 3, and 4), influenza viruses (types A and B), and respiratory syncytial virus. The remaining 20% of RTI episodes are caused by Group A β-haemolytic Streptococcus [2]. RTIs are a common cause of excessive or misuse of antibiotics that may induce antibiotic resistance—a pressing global public health concern.

Among RTIs, recurrent respiratory infections (RRIs) commonly occur in paediatric age, primarily affecting the upper airways, often emerging in preschool children within the initial six years of life when attending nursery school. Approximately 25% of infants under one year and 6% of children within the first six years’ experience recurrent respiratory infections [3]. Specific respiratory diseases, such as infectious rhinosinusitis and acute otitis media, have well-defined recurrence criteria. However, there is no international consensus on the number of respiratory infections considered pathological for a child. A recent Italian Consensus [3] suggests that a child aged 3 to 6 years with five or more respiratory tract infections (including one potentially severe pneumonia) in one year or two non-serious pneumonias confirmed by clinical and/or radiological criteria within a year may be classified as suffering from RRIs. These infections pose a significant medical challenge, especially in young children, leading to substantial morbidity and frequently causing school absences and medical visits.

Lactoferrin (Lf) is a natural iron-binding glycoprotein belonging to the transferrin family protein organized into two highly structured homologous lobes, the N-lobe and the C-lobe with each lobe able to bind to a single ferric ion (Fe^3+^). It is found primarily in the breast milk of all mammals but is also synthesized by exocrine glands and neutrophils, is abundantly present in all human biological fluids, such as tears, saliva, mucus, bronchial, gastric, bile, seminal and vaginal secretions [4]. In breast milk, Lf concentrations are at their highest in colostrum, gradually decreasing in mature milk, and remains relatively constant from one month to two years of lactation [5]. Furthermore, neutrophils also carry Lf in their secondary granules, and neutrophil degranulation releases Lf into plasma during systemic infection and inflammation [6,7].

Lf is involved in various biological processes such as the regulation of iron levels in biological fluids and immunoregulation. The antimicrobial and antiviral properties of Lf are well-known [8]. In the last two years, there has been accumulating evidence from studies in vitro and in vivo that Lf is active against SARS-CoV-2 [9,10,11]. Bovine lactoferrin (bLf), commercially available and used for years in paediatric settings, exhibits high structural homology with human lactoferrin [12]. In many in-vitro and in-vivo studies, bLf has demonstrated actions equivalent to human Lf [13]. Considered safe, bLf holds a “generally recognized as safe” (GRAS) status [14], allowing its global marketing as a nutritional supplement.

Several randomized controlled trials (RCTs) have highlighted the role of Lf administration in counteracting RTIs [8,15,16]. A recent meta-analysis [12], which included three trials involving adult subjects and six focusing on infants or toddlers, demonstrated that the administration of Lf effectively reduced the risk of RTIs. It also played a beneficial role in managing symptoms and facilitating the recovery of patients with RTIs. However, another meta-analysis conducted by Berthon et al. [16] indicated that while the incidence of RTIs was reduced in infants and children, there was no similar effect observed in adults.

Until now, all randomized RCTs involving children considered those from newborns up to 32 months old. There is a lack of studies focusing on preschool children and particularly those addressing RRIs. To date, only one study has explored the effects of bLf in combination with curcumin on reducing the frequency of RRIs [17].

To address this unmet need, the aim of this study was to investigate the impact of bLf daily oral administration in an Italian cohort of preschool children diagnosed with RRIs in the previous 12 months, in reducing the episodes of such infections and the use of medical treatment during the study period.

## 2. Methods

### 2.1. Study Design

This was a randomized trial designed to evaluate the efficacy of bLf in treated children compared to a control population (without bLf). The study was conducted from October 2022, covering autumn-winter seasons. The active phase (treatment phase), spanned 4 months, followed by a 2-month follow-up. The enrolment of patients in the study involved the participation of 19 primary care paediatricians.

The study was approved on 28/10/2022 by the Ethics Committee per le Sperimentazioni Cliniche della Provincia di Vicenza, Italy (Protocol number 113083) and parents or guardians provided written informed consent. This study was performed in accordance to the ethical standards laid down in the 1975 Declaration of Helsinki.

### 2.2. Patients

The study involved children aged between 3- and 6-years attending nursery/preschool, with a history of RRIs. A case of IRR was defined in our study as a frequency of 5 or more episodes of RRI in the previous season as defined by the recent Italian Consensus [3]. The children were regularly followed by their treating paediatricians.

Inclusion criteria encompassed children diagnosed with RRI in the previous year, while exclusion criteria comprised allergies to milk proteins, pathologies (primary or secondary immunodeficiency, cystic fibrosis, respiratory malformations, ciliary dyskinesia); ongoing immunostimulant treatment, and refusal to participate.

### 2.3. Calculation of Sample Size

The sample size was calculated by assuming a 30% reduction of the number of respiratory infections (primary endpoint) over a 4-month period in the active arm vs. the control arm. By enrolling 50 patients (allocation ratio 1:1) provides >80% power to detect as statistically significant (alpha error = 0.05) the expected between-arms difference of the primary endpoint. The sample size calculation also included a 25% potential attrition rate. All patients were centrally randomized, and the allocation sequence was concealed from those assigning participants to the intervention groups, until the moment of assignment to avoid selection bias.

### 2.4. Treatment

Patients in the treatment arm (active arm) received bLf in orodispersible capsule form at a daily dose of 400 mg/day (2 capsules of Mosiac^®^—Pharmaguida; each capsule contained 200 mg of bLf), divided into two administrations, for 4 months, away from meals. In the case of an ongoing infection, the administration of bLf continued without interruption alongside the prescribed pharmacological treatment, as judged by the paediatrician.

Patients in the control group did not receive bLf, and in the event of an infection, they were treated with the prescribed pharmacological treatment, as determined by the paediatrician. The use of immunostimulant products was not considered during the course of the study.

### 2.5. Outcome Measures

The primary objective of this study was to evaluate the impact of bLF supplementation on respiratory infection episodes during the 4-month study period, comparing treated and untreated groups. Secondary objectives included assessing treated and untreated groups for episodes requiring antibiotic and/or inhaled/oral corticosteroid treatment, days with symptoms, and days of school absence.

### 2.6. Statistical Analysis

Data were summarized as mean and standard deviation (SD), median and interquartile range (IQR), or as absolute number and percentage, and the comparisons between two groups were performed by unpaired T-Test, Mann-Whitney U test, or Chi Square Test, as appropriate. Data analysis was performed according to the intention to treat (ITT) principle. The effect of bLF on the incidence of respiratory infections was also investigated by ordinal logistic regression analysis having as dependent variable respiratory infections grouped as follows: group 1, no episode; group 2, 1–2 episodes; group 3, ≥3 episodes. In the ordinal logistic regression analysis, the proportionality of odds was formally tested by the likelihood ratio test (LR test) and data were expressed as odds ratio (OR), 95% confidence interval (CI), and *p* value. The impact of bLf on the incidence of recurrent respiratory infections (≥3 episodes) and of corticosteroid use was assessed by calculating the number needed to treat (NNT). Data analysis was carried out by STATA 16, StataCorp, Lakeway Drive, College Station, TX, USA.

## 3. Results

### 3.1. Patient Characteristics

The study sample included 50 children randomly allocated to the active arm (*n* = 25, on bLf, 400 mg/day divided in two administrations, for 4 months) and the control arm (*n* = 25) of the trial. All attended nursery school during the study period. Patients were enrolled between October and the beginning of December 2022. Overall, their mean age was 4.2 ± 0.1 years, 32 were males (64%) and the majority (*n* = 39, 78%) had one or more respiratory symptoms at enrolment (fever in 7 cases, cough in 32 cases, sore throat in 15 cases, ear pain in 4 cases, nasal congestion in 26 cases, runny nose in 18 cases, and bronchospasm in 3 cases). Fourteen families had at least one smoker among the cohabiting family members and the median number of family members was 4.

At baseline, 9 patients were treated with antibiotics, 16 with anti-pyretics, 3 with mucolytics, 16 with cortisone by aerosol, 6 with oral cortisone, and 5 with bronchodilators. Before enrolment, the median number of days with symptoms and of absence from school was 4 and 3, respectively. The two groups were similar with regard to demographic and clinical variables (Table 1).

### 3.2. Effect of bLf on Frequency of Respiratory Infections

**Figure 1:**
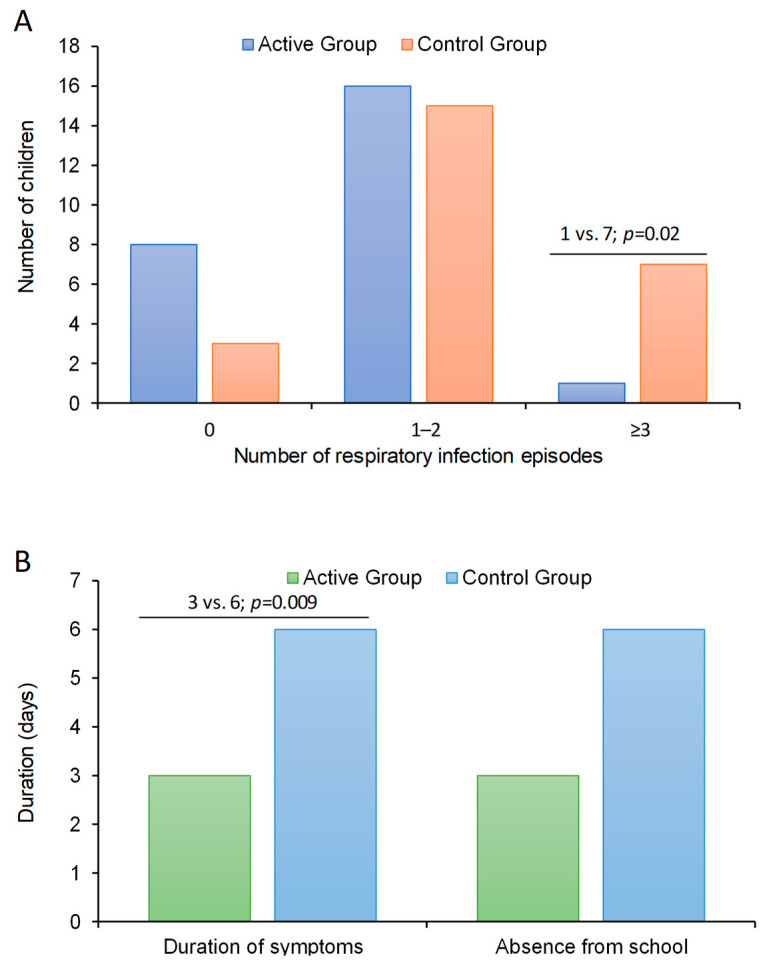
Results from primary and secondary outcomes in preschool children supplemented with lactoferrin vs. control. (**A**), primary outcome; number of respiratory infection episodes in the two arms and (**B**), secondary outcomes, duration of symptoms and days absent from school in the two arms.

During the active phase of the trial, the median number of episodes of respiratory infections was 50% lower (*p* = 0.02) in children treated with bLf (median: 1 episode, IQR:0–2) than in those untreated (median: 2, IQR: 1–3) (Table 2). The proportion of participants with a number of infection episodes ≥3 was significantly lower (*p* = 0.02) in the active arm (*n* = 1, 4%) than in the control arm (*n* = 7, 28%) with a number needed to treat of 4. This implies that every 40 children treated with bLf for 4 months, 10 cases of recurrent infections (≥3 episodes) were avoided vs. those of the control group. Across the active phase of the trial, 11 patients had no respiratory infections (8 in the active arm and 3 in the control arm), 31 patients had 1–2 respiratory infections (16 in the active arm and 15 in the control arm), and the remaining 8 patients had ≥3 respiratory infections (1 in the active arm and 7 in the control arm) (Figure 1A). In an ordered logistic regression analysis having the number of episodes of respiratory infections during the active phase of the trial as dependent variable (no episode; 2–3 episodes; and ≥3 episodes), the odds ratio of having a higher number of infection episodes over time was 80% lower in children of the active arm than in those of the control arm (OR: 0.20, 95% CI: 0.06–0.74, *p* = 0.015; LR test of proportionality of odds: χ^2^ = 0.71, *p* = 0.40).

### 3.3. Secondary Outcome Measures and Follow Up

The duration of symptoms was significantly lower (*p* = 0.009) in patients of the active arm (median: 3 days, IQR: 0–6) than in those of the control arm (median: 6, IQR: 5–9) (Figure 1B). Accordingly, the days of absence from school was lower in bLf treated patients (median: 3 days, IQR: 0–7) than in those untreated (median: 6 days, IQR: 2–8) even if this difference did not attain the statistical significance (*p* = 0.15) (Table 2).

During the 2-month follow-up period no between-arms difference was found as for the number of episodes of respiratory infections, the duration of symptoms, and the days of absence from school (Table 2). The proportion of patients on treatment with corticosteroids over the whole study period (6 months) was significantly lower in the active arm than in the control arm of the trial (32% vs. 60%; *p* = 0.047) (Table 2). This implies that for every 60 children treated with bLf for 4 months, 10 patients needing cortisone are avoided vs. those in the control group. No difference between the two groups was found as for the use of antibiotics. The proportion of patients with fever, COVID-19, and who underwent to flu vaccination did not differ between the two groups (Table 2). No adverse events were observed across the study period in both the active and the control arm.

## 4. Discussion

To the best of our knowledge, this is the first RCT demonstrating the efficacy of bLf supplementation on RRI in preschool children. We observed a clinically relevant 50% reduction in respiratory infection episodes during the active phase of the trial, with a number needed to treat of 4. Furthermore, treated patients exhibited an 80% reduction in the odds of experiencing multiple episodes, coupled with shorter symptom duration compared to the untreated group (median of 3 vs. 6 days, respectively). Although not achieving statistical significance, it is important to note that the days of absence from school were lower in the bLf-active arm (median: 3 days) than in untreated group (median: 6 days, *p* = 0.15).

The 2-month follow-up period revealed no significant differences between the two arms in terms of episodes of respiratory infections and duration of symptoms. Over the entire 6-month study duration, a significant reduction in the use of corticosteroids was observed in the bLf-treated group vs. control group. No differences were observed in antibiotic use, fever, COVID-19 incidence, or flu vaccination between the groups.

Supporting our findings, a recent meta-analysis by Ali et al. [12] demonstrated the potential of bLf in reducing the risk of RTIs in both adults and children. Another meta-analysis by Berthon et al. [16], however, showed that RTI incidence was reduced only in infants and children (OR: 0.78; 95% CI: 0.61, 0.98) and not in adults (OR: 1.00; 95% CI: 0.76, 1.32). There are four existing RCTs evaluating the effect of Lf in RTIs in children, also considered in the previously mentioned meta-analyses, predominantly focused on infants and toddlers ([18,19,20,21]). Chen et al. [19] reported results from a multicentre study conducted in China, with a total of 260 infants aged 4–6 months, randomized into a lactoferrin-fortified formula milk group (containing lactoferrin 38 mg/100 g milk) and a no lactoferrin-fortified milk group. The intervention lasted for 3 months. They found a decreased incidence of overall respiratory-related illness, including rhinorrhea, cough, wheezing, or nasal congestion. King et al. [20] enrolled 52 healthy, formula-fed infants in a paediatric clinic in the United States, all less than 4 weeks of age. Infants received either formula supplemented with lactoferrin (850 mg/L) or commercial cow milk–based formula (102 mg/L) for 12 months. Their results showed a decreased incidence of lower RTIs, primarily wheezing, in infants who received formula supplemented with lactoferrin (850 mg/L) compared with controls who were given a cow milk–based formula fortified with 102 mg/L of bLf for 12 months of intervention. In the study by Li et al. [18], performed in China, 451 healthy term infants were enrolled from 3 clinical sites and randomized to a cow’s milk-based infant formula or milk fat globule membrane (MFGM) + Lf (a similar infant formula, with an added source of bovine milk fat globule membrane [bMFGM; whey protein-lipid concentrate, 5 g/L] and bLf [0.6 g/L]) through 365 days of age. The active phase lasted for 12 months, while the follow-up continued for an additional 18 months. The overall incidence of respiratory-associated adverse events was significantly lower for the MFGM + Lf group through day 545.

In a prevalence study undertaken in Japan, Motoki et al. [21] evaluated the occurrence of acute respiratory symptoms in 101 children aged 12 to 32 months randomized into the placebo arm or bLf (48 mg/day)-fortified formula (active arm) for 13 weeks. During the intervention period, they reported a significantly lower duration (total number of days) in acute respiratory symptoms in the bLf group compared to the placebo group. Taken together, data derived from these RCTs are in line with our results, showing a 50% reduction in respiratory infection episodes, translating to a number needed to treat of 4. However, these studies were conducted on infants and toddlers, while ours was focused on preschool children. To our knowledge, only one uncontrolled study evaluating the effect of Lf supplementation in preschool children has previously been performed [17], with an age very close to that of the patients in our trial. Furthermore, in this study (as well as ours), children with a diagnosis of RRIs were considered. In this trial, 10 children (3–7 years of age) were all supplemented with 900 mg/day of bLf and 100 mg/day of curcumin from October to February [17]. Zuccotti et al. found that Lf supplementation in children with RRIs was associated with a beneficial immunomodulatory effect. Even if they did not report the clinical impact of bLf in children with RRI, their findings could explain our results showing an 80% reduction in the odds of experiencing multiple episodes, implying that out of 40 children treated with bLf for 4 months, 10 cases of recurrent infections (≥3 episodes) were avoided compared to the untreated group.

Regarding the duration of symptoms, we observed a shorter duration of symptoms in children supplemented with bLf compared to the untreated group (medians: 3 days vs. 6 days, respectively). Three trials have reported either the cumulative duration (total number of days affected) or the mean duration of episodes of respiratory illnesses or RTIs, with two trials [19,21] reporting a reduction, and one trial [20] reporting no difference in bLf-supplemented subjects vs. controls. A reduction in the duration of runny nose episodes but no effect on the duration of wheezing or cough episodes was reported in a 3-month intervention with 38 mg/100 g bLf-fortified infant formula in healthy infants [19]. In addition, in healthy children given 48 mg/day bLf fortified formula for 13 weeks, a decrease in the cumulative duration of respiratory illness was observed but no effect on episode duration was reported [21].

No study has specifically focused on the impact of bLf in reducing school absenteeism due to respiratory tract infections (RTIs). According to our data, the number of days absent from school was lower in bLf-treated patients (median: 3 days, IQR: 0–7) compared to those untreated (median: 6 days, IQR: 2–8), although this difference did not reach statistical significance (*p* = 0.15).

The reduction in cortisone requirement (64% vs. 48% in the active arm compared to the control group) observed in our study was particularly relevant. The sustained decrease in cortisone use over the entire study period suggests potential long-term benefits, highlighting the enduring impact of bLf supplementation on reducing the need for cortisone interventions. This specific observation represents the initial occurrence in which bLf supplementation has been associated with a reduced need for cortisone treatment, as no studies exploring this connection are available, and indicates a potential avenue for minimizing the need for immunosuppressive treatments, suggesting an improved immune function induced by bLf. Its multifaceted protective effects include antibacterial, antiviral, and immunomodulatory activities [15,22]. Indeed, studies demonstrate LF’s effectiveness against a range of viruses in vitro and in vivo, showcasing its potential in the management of viral infection [8].

During the COVID-19 epidemic, orally-administered bLf has shown promising results in reducing viral clearance times and symptom duration in SARS-CoV-2-infected patients [9]. Indeed, Lf is recognised as a protein of the innate immune system, as it bridges innate and adaptive immunity by recruiting leukocytes and activating dendritic cells [23,24]. It stimulates T and B lymphocytes, enhancing both cellular and humoral immune responses [25,26]. LF’s anti-inflammatory action involves the regulation of pro-inflammatory gene expression, reducing cytokine synthesis, and promoting nonspecific immune responses [27].

In addition, in the present study, as per exclusion criteria, primary immunodeficiencies were not tested with dosages of immunoglobulins and other immunological parameters. It is important to stress that all children included in this study were attending nursery/preschool and therefore a high number of RRIs was to be expected. In addition, no children presented atypical infections with onset in the first months of life, that is important to suspect a primary immunodeficiency disease [28,29].

## 5. Study Limitations

Some weaknesses of our study need to be mentioned. This was a study conducted in a single region of the north of Italy, potentially limiting the generalizability of our findings to a broader population. Future multi-center studies could offer a more comprehensive understanding of the effectiveness of lactoferrin supplementation across diverse geographical and demographic settings. Moreover, we employed a 2-month follow-up period that revealed no significant differences between the bLf-treated group and the control group. The relatively short duration may not capture potential long-term effects or fluctuations in respiratory infection patterns over an extended period. Longitudinal studies with extended follow-up periods could provide valuable insights into the sustained efficacy of lactoferrin.

Finally, our study did not extensively evaluate the presence of comorbidities or underlying health conditions that might influence susceptibility to respiratory infections. Future studies should consider a more detailed assessment of participants’ health status to better understand the other clinical characteristics affecting outcomes.

## 6. Conclusions

Our study provides valuable insights into the potential benefits of bLf supplementation in reducing respiratory infection episodes in preschool children. Addressing the limitations above mentioned in future research will contribute to a deeper comprehension of the role of bLf in paediatric respiratory health.

## Figures and Tables

**Table 1:** Baseline clinical characteristics of patients enrolled in the study in the whole group and stratified by the two study arms.

	Whole Group (*n* = 50)	Control Group (*n* = 25)	Active Group (*n* = 25)	*p*-Value
Age (years)	4.2 ± 0.1	4.4 ± 0.2	4.0 ± 0.2	0.14
Male gender, *n* (%)	32 (64)	14 (56)	18 (72)	0.24
Number of families with at least one member who smokes, *n* (%)	14 (28)	7 (28)	7 (28)	1.00
Number of family members, *n*	4 (3–4)	4 (3–4)	4 (3–4)	0.17
*Symptoms*				
respiratory, *n* (%)	39 (78)	20 (80)	19 (76)	0.73
fever, *n* (%)	7 (14)	5 (20)	2 (8)	0.22
cough, *n* (%)	32 (64)	16(64)	16(64)	1.00
sore throat, *n* (%)	15 (30)	8(32)	7(28)	0.76
ear pain, *n* (%)	4 (8)	3(12)	2(4)	0.30
nasal congestion *n* (%)	26 (52)	12 (48)	14 (56)	0.57
runny nose, *n* (%)	18 (36)	10 (40)	8 (32)	0.56
bronchospasm, *n* (%)	3 (6)	1 (4)	2 (8)	0.55
*Treatment*				
antibiotic, *n* (%)	9 (18)	4 (16)	5 (20)	0.71
anti-pyretic, *n* (%)	16 (32)	9 (36)	7 (28)	0.54
mucolytic, *n* (%)	3 (6)	2 (8)	1 (4)	0.55
corticosteroids (aerosol), *n* (%)	16 (32)	7(28)	9 (36)	0.54
Oral corticosteroids, *n* (%)	6 (12)	4(16)	2(8)	0.38
bronchodilators *n* (%)	5 (10)	2 (8)	3 (12)	0.64
Duration of symptoms (days)	4 (2–6)	4 (3–7)	4 (1–5)	0.29
Number of days of absence from school	3 (0–5)	3 (2–5)	1 (0–5)	0.37

Data are reported as mean ± SD, median and interquartile range, or absolute number and percentage, as appropriate.

**Table 2:** Study outcomes in the control group compared to the active group.

	Control Group (*n* = 25)	Active Group (*n* = 25)	*p*-Value
*4-month period (active phase)*			
Episodes of respiratory infections (*n*)	2 (1–3)	1 (0–2)	0.02
Duration of symptoms (days)	6 (5–9)	3 (0–6)	0.01
Absence from school (days)	6 (2–8)	3 (0–7)	0.15
*2-month period (follow-up)*			
Episodes of respiratory infections (*n*)	0 (0–1)	1 (0–1)	0.23
Duration of symptoms (days)	0 (0–4)	0 (0–3)	0.84
Absence from school (days)	0 (0–4)	0 (0–0)	0.60
*Total period*			
Treated with antibiotics, *n* (%)	9 (36)	12 (48)	0.39
Treated with cortisone, *n* (%)	15 (60)	8 (32)	0.047
Patients with fever, *n* (%)	16 (64)	12 (48)	0.25
Patients with COVID, *n* (%)	2 (8)	1 (4)	1.00
Patients with flu vaccine, *n* (%)	9 (36)	9 (36)	1.00

Data are reported as median and interquartile range, or absolute number and percentage.

## Data Availability

Data is contained within the article.
